# Home is where the ad is: online interest proxies housing demand

**DOI:** 10.1140/epjds/s13688-018-0176-2

**Published:** 2018-11-09

**Authors:** Marco Pangallo, Michele Loberto

**Affiliations:** 1 Institute for New Economic Thinking at the Oxford Martin School, University of Oxford, Oxford, United Kingdom; 2 Mathematical Institute, University of Oxford, Oxford, United Kingdom; 3 Directorate General for Economics, Statistics and Research, Banca d’Italia, Roma, Italy

**Keywords:** Online data, Housing market, Econometrics, Machine learning, Causality

## Abstract

Online activity leaves digital traces of human behavior. In this paper we investigate if online interest can be used as a proxy of housing demand, a key yet so far mostly unobserved feature of housing markets. We analyze data from an Italian website of housing sales advertisements (ads). For each ad, we know the timings at which website users clicked on the ad or used the corresponding contact form. We show that low online interest—a small number of clicks/contacts on the ad relative to other ads in the same neighborhood—predicts longer time on market and higher chance of downward price revisions, and that aggregate online interest is a leading indicator of housing market liquidity and prices. As online interest affects time on market, liquidity and prices in the same way as actual demand, we deduce that it is a good proxy. We then turn to a standard econometric problem: what difference in demand is caused by a difference in price? We use machine learning to identify pairs of duplicate ads, i.e. ads that refer to the same housing unit. Under some caveats, differences in demand between the two ads can only be caused by differences in price. We find that a 1% higher price causes a 0.66% lower number of clicks.

## Introduction

Online activity makes it possible to quantify aspects of human behavior that were not previously measurable at a comparable scale. Examples include stock market sentiment [1, 2], ideological conflict [3, 4], social networks [5, 6], mobility [7] and epidemic spreading [8]. In this paper we quantify housing demand, as viewed through the lenses of online activity on an Italian website of housing sales advertisements (ads). We first establish that online interest is a good proxy of actual demand, and then, on a more technical level, we combine econometric and machine learning ideas to investigate the causal link from prices to demand.

The interaction between housing demand and supply determines price trends and the social composition of neighborhoods. Higher demand—if supply does not increase—is associated with increasing prices and consequently worsening residential income segregation. This insight can easily be formalized in various types of models of the housing market: spatial equilibrium models [9, 10], search and matching models [11–13] and agent-based models [14–16].

However, empirically testing the effect of demand is much harder, because demand is hard to measure. For example, Genesove and Han [17] write “however as buyers are not listed in North American housing markets, the stock of them is impossible to construct for empirical work.” Genesove and Han use changes in income and population at the city level as proxies of demand [17]. Carrillo *et al.* [18] use seller bargaining power and sale probability. Merlo and Ortalo-Magné [19] analyze what is arguably the most complete dataset in terms of demand information. Their data include the number and timing of viewings to listed properties and the sequence of offers by potential buyers. However, data are hand-collected by the agencies, limiting their sample size to 780 units.

The advent of the internet has made it possible to quantify demand on a larger scale. Potential home buyers start gathering information about dwellings by browsing the internet, and may subsequently contact an agency to obtain more detailed information or organize a viewing. Wu and Brynjolfsson [20] are the first to use internet data to quantify demand, showing that the number of Google housing-related searches is predictive of future price appreciations and higher volume of transactions at the city level. (See also Ref. [21] for Google searches.) Van Dijk and Francke [22] come to the same conclusion, but their measure of demand is the aggregate number of clicks on housing ads on a Dutch website, where aggregation is performed again at the city level.

Here we go one step further and analyze measures of online interest *at the level of individual ads*. We have access to the full temporal sequence of the number of clicks on each ad, from the time the ad was posted to the time it was removed from the website. We also know the timings in which potential buyers used the contact form on the website to contact sellers. We show that our measures of online interest are predictive of the time on market and of the probability and magnitude of both downward and upward price revisions. We also aggregate the number of clicks and contacts at the neighborhood and city level, and confirm the results in Refs. [20, 22] in terms of liquidity and price trends. As time on market, liquidity and prices are linked to actual demand in the same way as our measure of online interest, we deduce that clicks and contacts are a good proxy of actual demand.

The main problem with our dataset is the large fraction of duplicate ads, namely multiple ads that refer to the same dwelling. For example, an agency might post a new ad for the same dwelling to make the new ad appear at the top of “most recent” listings, without deleting the old ad. It is clear that if one does not deal with the existence of duplicates, results on time on market and price revisions are likely to be biased. To address this issue, we devise a machine learning algorithm that identifies duplicates. We use a classification tree with boosting to assign to pairs of ads probabilities to be duplicates, and consider pairs with probability larger than 0.5 as duplicates.

The identification of duplicate ads is also very useful to estimate the *price elasticity of demand*. This is the relative difference in demand—in this case, the number of clicks—that is caused by a relative difference in price. (Clarification: a price revision for an ad and the price elasticity of demand are used as different concepts here. A price revision means that the ad was already online when the price change occurred. The price elasticity of demand is more of a thought experiment: had the ad been posted with a different price, what would the relative difference in clicks be?) The elasticity of demand is an extremely important concept for both businesses and policy. Many companies need to know how changing prices would affect the demand for their goods, and many institutions need causal understanding of the link between prices and demand to implement some policies. For example, a city council may want to start a program of housing subsidies. By subsidizing poor households this policy effectively decreases the price of houses for those households, and its success depends on the effect on demand.

A regression in which the dependent variable is the number of clicks and the independent variable is the price yields an incorrect estimate of the elasticity, mainly because both the price and the number of clicks are correlated with other variables, such as the intrinsic quality of the dwelling or of the neighborhood. But if the dwelling is the same and only the price of the corresponding duplicate ad is different—for example, because the agency posted a new ad with a different price—the elasticity can be estimated consistently from pairs of ads. There are some caveats. For example, users must not be able to identify duplicates before clicking on them (given the way the search engine of our website works, we think that this is reasonable in most cases).

With this approach we relate to the literature on demand *identification* [23–25], whose goal is to understand the causal structure of demand using exogenous demand and supply shocks. A recent work in this literature uses the Uber pricing system to identify the full demand curve [26]. Given data constraints, here we only identify the demand elasticity, and we use an imperfect proxy of demand such as the number of clicks rather than considering realized demand. A rather different literature is concerned with demand *forecasting* [27]:^1^ given a set of house and neighborhood characteristics, what is the most accurate prediction of the house demand? As demand forecasting is purely a prediction task, machine learning algorithms are likely to perform best (see e.g. the Zestimate Competition at https://www.kaggle.com/c/zillow-prize-1). Demand identification requires instead causal reasoning that is usually formalized in terms of econometric techniques. Cross-fertilization between machine learning and econometrics has been advocated recently [28], and has already delivered a substantial amount of research [29, 30]. Our contribution in this direction combines ideas from classification in supervised machine learning and the potential outcomes framework [31, 32] in statistics and econometrics. Our method can generally be applied to any marketplace website and not necessarily to housing demand.

The rest of this paper is organized as follows. In Sect. 2 we describe the data; in Sect. 3 we provide descriptive statistics on the temporal and spatial aspects of clicks and contacts, both at the level of individual ads and aggregating over neighborhoods and cities. In Sect. 4 we provide evidence that online interest is indeed a proxy of housing demand, and in Sect. 5 we introduce the methodology to estimate the price elasticity of demand. Section 6 concludes.

## Data

**Figure 1:**
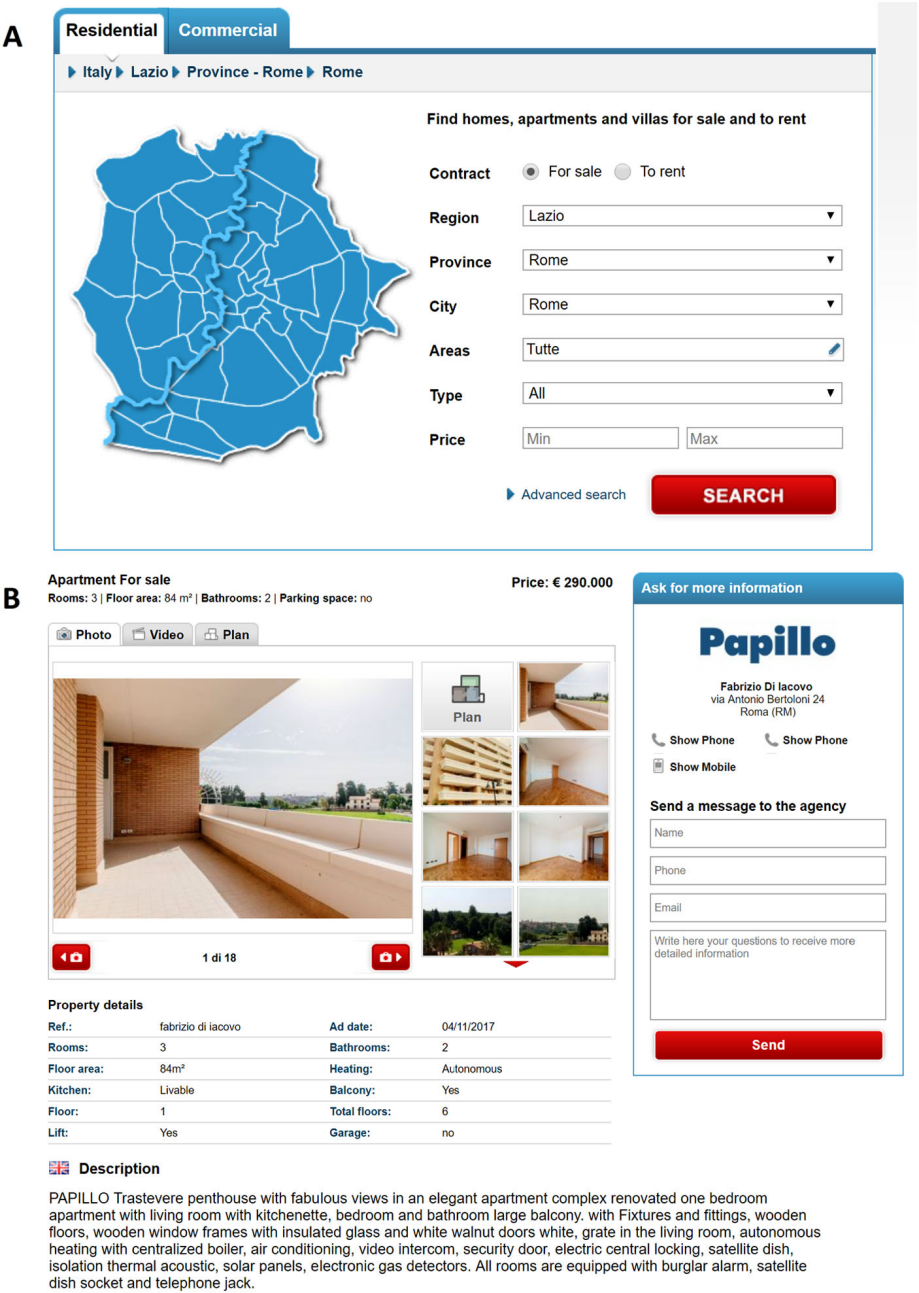
Screenshots from the webpage of Immobiliare.it (English version). (**A**) Search engine. (**B**) Sample ad. The form to contact the agency is on the right

We analyze a unique dataset provided by Immobiliare.it (www.immobiliare.it), the largest website of housing ads in Italy. Website users look for a dwelling by specifying a location, a price range and other criteria (Fig. 1A). The website returns a list of ads, and the users can click on any ad to obtain more detailed information (Fig. 1B). If they are interested, users can contact the real-estate agency by phone, or using the form that is provided on the website.

Our data consist of multiple snapshots of the Immobiliare.it database, from January 2015 to June 2017. By snapshot we mean all information on ads that are visible on a specific day. For 2015 we only have quarterly snapshots, while from 2016 on we mostly have weekly snapshots. In practice, most ads remain unchanged between two weekly snapshots, with about 5% of the ads being removed and 5% being newly uploaded. We retain time-varying information for the variables we are mostly interested in—(asking) price, number of clicks on the ad, and number of contacts that occurred through the website. (There is a counter of clicks and contacts that increases over successive snapshots.)

For other variables we instead rely on the latest available information, because we assume that the sellers correct the mistakes they might have made when posting the ad. These variables are the physical characteristics of the dwelling—floor area, number of rooms, maintenance status, etc. (see Fig. 1B)—and its geographical coordinates. We are also given a brief description of the dwelling. This description tends to contain the same information that is stored in the other variables, but also provides more details about the neighborhood and the agency that sells the property. Finally, we know the dates in which the ad was uploaded and in which the ad was removed (if it was).

In this paper we only focus on residential units for sale in the 110 province capital cities, which include all major cities and comprise about 18 million inhabitants in total. In cities the majority of transactions is brokered by real estate agents—who are more likely to upload an ad on Immobiliare.it than private citizens—, whereas in small towns and in rural areas representativeness is potentially a problem. The set of ads we will work on encompasses 1,037,095 units.

However, not all ads refer to a distinct dwelling. Indeed, there is a substantial fraction of *duplicate* ads, that is two or more ads that refer to the same dwelling. The existence of duplicates is due to several reasons. First, in Italy there is no legal obligation for owners to entrust at most one real estate agent for the sale of their property. This means that two or more real estate agents may be selling the same dwelling at the same time. Second, the same agency may remove an ad and upload an identical one, so that the new ad is more recent. (In Sect. 3.1 we show that most clicks on any ad occur within the first few days the ad is posted.) Third, the mandate of an agency may cease and the seller could decide to entrust another agency, which would then upload a new ad. In previous work [33] we showed that the existence of duplicates is not random and that keeping duplicate ads can lead to a serious misrepresentation of the supply of dwellings for sale, especially when looking at small geographical aggregates. We identify duplicate ads using a machine learning methodology, described in Sect. 5.1 and in Ref. [33] in much more detail. According to our procedure, the total number of dwellings is 653,499 units, about 63% of the total number of posted ads.

Finally, we use the geographical coordinates of the ads to match our data with two administrative datasets. The first comes from Osservatorio del Mercato Immobiliare (OMI), the real-estate market observatory of the Italian Tax Agency. From this dataset we extract the perimeters of the so-called OMI microzones, homogeneous areas in terms of socioeconomic and geographic characteristics that roughly correspond to neighborhoods.^2^ We then perform spatial matching and assign to each ad its corresponding OMI microzone. The second dataset is the Italian 2011 Census, providing information on socioeconomic characteristics. As census tracts do not correspond to OMI microzones, we impute data to OMI microzones depending on the percentage of overlap between each census tract and OMI microzone (see Ref. [33] for more details).

## Descriptive statistics on clicks and contacts

We quantify online interest by the number of clicks and contacts. For each ad and every snapshot of the database, we record new clicks/contacts, so as to follow the full evolution of these variables over the lifetime of all ads. We perform our analysis both at the level of individual ads, and after aggregation at the OMI microzone/city level. In this section we provide some descriptive statistics on clicks and contacts for all spatial aggregation levels.

### Individual statistics

We only focus on ads uploaded from 2016 (since we only have quarterly snapshots in 2015) and subsequently removed from the dataset, to make sure that we follow all the lifetime of the ads. This corresponds to 329,915 ads.

**Figure 2:**
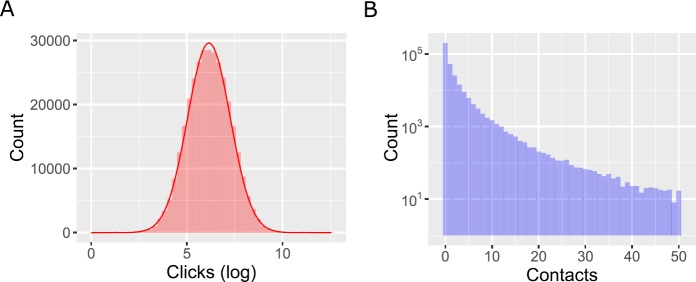
Distribution of the total number of clicks and contacts throughout the lifespan of ads. (**A**) The total number of clicks almost perfectly follows a log-normal distribution (red solid line). (**B**) The total number of contacts follows a heavy-tailed distribution, but only varies over two orders of magnitude

First of all, we analyze the distribution of the total number of clicks and contacts throughout the lifespan of ads (Fig. 2). The median number of clicks is 468, whereas the mean is 861, suggesting that the number of clicks follows a heavy-tailed distribution. In particular, we check if this quantity follows a log-normal distribution (red solid line in Fig. 2A). The fit is very good, but a Kolmogorov–Smirnov test formally rejects this hypothesis (*p*-value = 3.706 × 10^−13^). Indeed, due to the large sample size even a small deviation between the empirical and log-normal cumulative distributions is likely to be statistically significant. The excess kurtosis is −0.044, suggesting that the distribution is platykurtic. However, a QQ-plot (not reported) shows that there are several outliers on the left tail—that is, there is a higher number of ads that received very few clicks as compared to what would be expected under a log-normal distribution.

In Fig. 2B we show a histogram with the total number of contacts (note the logarithmic scale on the vertical axis) that occurred through an ad. Each bin in the histogram corresponds to a unit, so that the first bin is the number of ads that received 0 contacts, etc. The median is 0 (201,934 ads received no contacts), suggesting that the contact form on the webpage is only used in a minority of cases. In case it is used, for most ads the contact form is used once (52,788), twice (25,569) or three times (14,206). However, the number of contacts decays slowly. The distribution is very heavy-tailed, and some ads received a large number of contacts. We do not attempt to identify the shape of this distribution as it varies over too few orders of magnitude (while the figure has a cutoff at 50 contacts, only 161 ads received between 50 and 100 contacts, and only 20 received more than 100 contacts. We suspect many of these could be outliers for which particular conditions apply).

**Figure 3:**
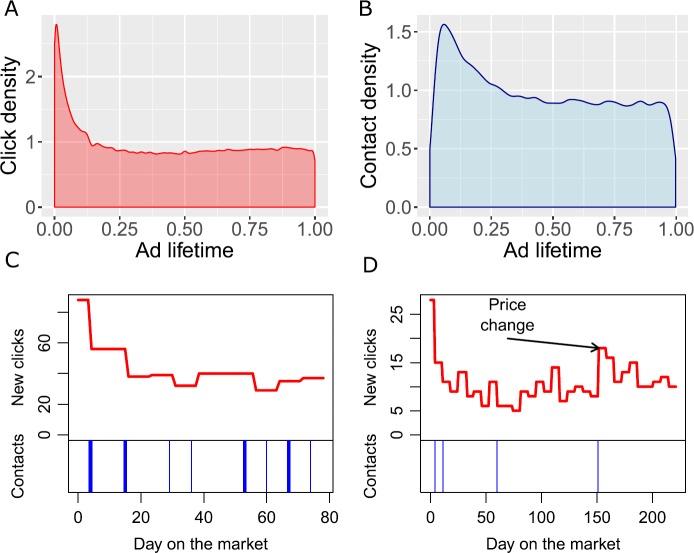
Temporal profile of clicks and contacts over the lifespan of ads. (**A**)–(**B**) Kernel density plots of the normalized times at which clicks/contacts occur. Both are peaked at the beginning of the ad lifetime, but the peak is more pronounced in the case of clicks. (**C**)–(**D**) Sequence of clicks/contacts for two sample (non-representative) ads. Clicks are normalized on a daily basis and the curves are discontinuous due to the fact that we only observe weekly snapshots of the database. In panel (**D**) there was a rise of clicks corresponding to a downward price revision. Contacts are shown as a barcode (the width of the bars is proportional to the contacts received in a week)

In Fig. 3 we follow the evolution of clicks/contacts over the lifespan of ads. In panels A and B we show the kernel density estimates of the times at which clicks/contacts occur, as normalized over the lifetime of the ad (the normalization is necessary to compare ads with different lifetimes). The highest frequency of clicks is in the first few days after the ad was uploaded, but clicks are distributed throughout the lifetime of the ad. Also contacts are peaked immediately after the upload, but the distribution is more uniform. In panels C and D we show the temporal evolution of clicks/contacts for two specific ads. These two ads are selected for visualization purposes and so are not representative. In panel D there was a downward price revision at approximately 2/3 of the lifetime of the ad. This price change lead to a spike in the number of clicks, although similar spikes occurred at other times too.

### Aggregate statistics

We consider the temporal and spatial distribution of clicks and contacts, as aggregated either at the level of cities or OMI microzones.

**Figure 4:**
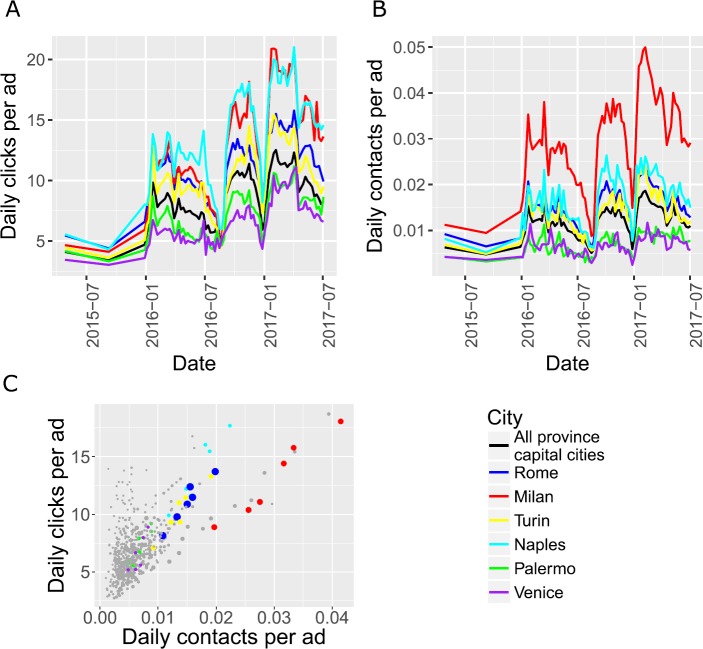
Clicks and contacts in the main Italian cities. (**A**)–(**B**) Temporal evolution of clicks and contacts in six cities and in the aggregate (solid black line). The time series are strongly cross-correlated and display seasonality. (**C**) Correlation between clicks and contacts. Each dot is a city in a quarter between 2016Q1 and 2017Q2. The size of dots is proportional to the number of visible ads, cities not shown in panels (**A**)–(**B**) are colored grey. The correlation coefficient is 0.64

In Fig. 4A–B we report the time series of daily clicks/contacts per ad in the main Italian cities and averaged over all province capital cities. The time series are highly cross-correlated—both among cities and between clicks and contacts. The series also display strong seasonality. Indeed, around Christmas and in July/August the number of clicks/contacts drops substantially (these are the most popular periods for vacation in Italy). In panel C we show the correlation between clicks and contacts. Here data are aggregated over cities and over quarters.^3^ The correlation coefficient is 0.64, suggesting good but not perfect correlation. For the six cities in panels A–B, we note that Milan and Naples have the highest number of clicks/contacts per ad. While both cities have a similar number of clicks per ad, Milan has many more contacts per ad than Naples. Rome and Turin are slightly above the average number of clicks/contacts in the province capital cities, whereas Palermo and Venice are well below average.

**Figure 5:**
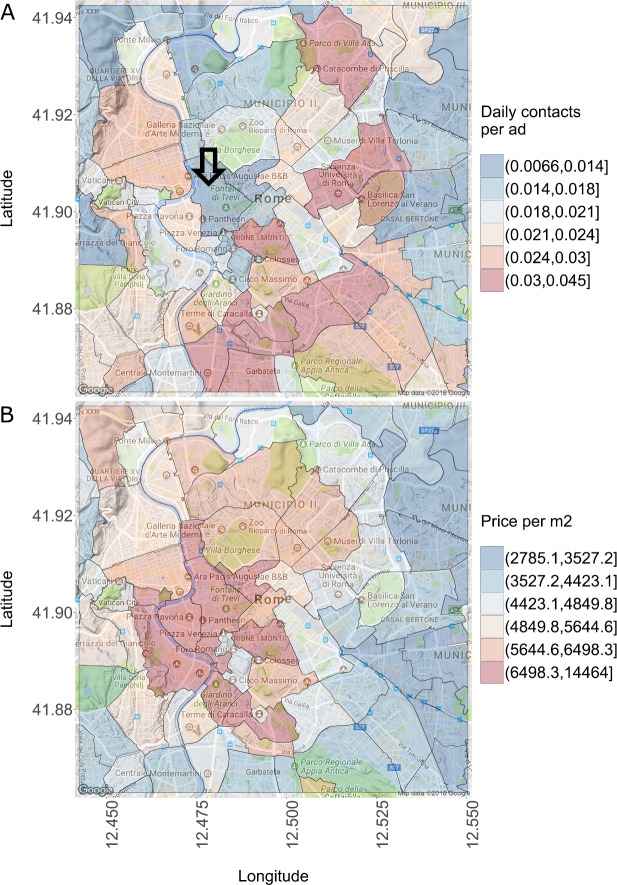
Contacts and price in Rome. Each polygon corresponds to an OMI microzone. (**A**) Daily contacts per ad, grouped in sixtiles. The arrow indicates one of the most touristic neighborhoods of Rome, which is however in the lowest sixtile of the contact distribution. (**B**) Price per m2, grouped in sixtiles. Prices are highest in the center, but higher in the North West than in the South East

**Figure 6:**
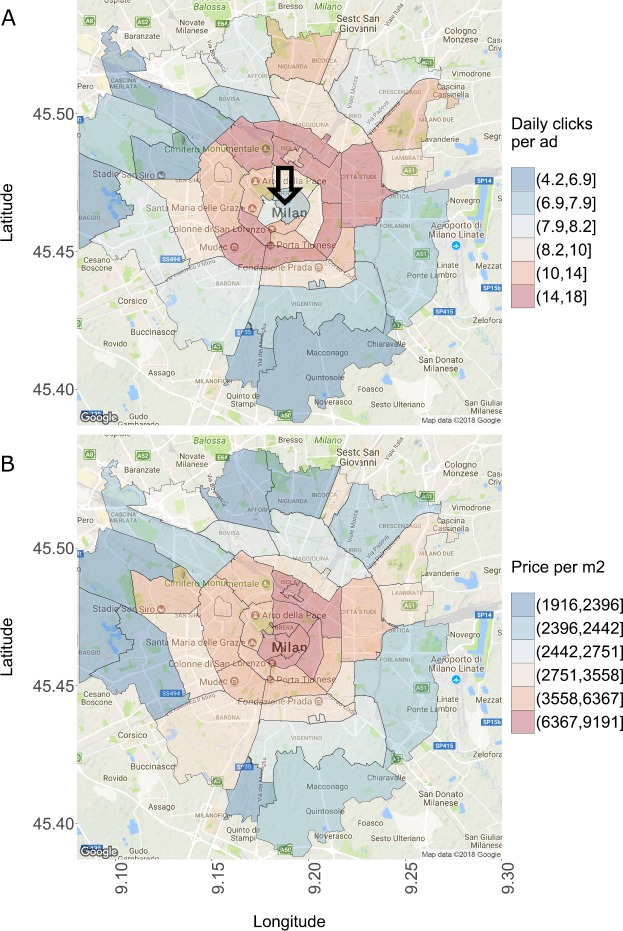
Clicks and price in Milan. Each polygon corresponds to an OMI microzone. (**A**) Daily clicks per ad, grouped in sixtiles. The arrow indicates the most central (and expensive) neighborhoods, in the lowest sixtiles of the clicks distribution. (**B**) Price per m2, grouped in sixtiles. Prices are highest in the center and decrease radially towards the peripheries

In Figs. 5 and 6 we zoom in from the point of view of geographical aggregation and compare clicks and contacts to the price per m2 in the OMI microzones of the two largest Italian cities, Rome and Milan, using data from the first quarter of 2016. For illustrative purposes we focus on contacts in Rome and on clicks in Milan (the patterns are similar across clicks/contacts). In Fig. 5A we see that the highest number of daily contacts per ad is South-East of the center (with the exception of the Monti neighborhood in the center). An arrow indicates one of the most touristic neighborhoods in Rome, where the Trevi Fountain and the Pantheon are located. This neighborhood is in the lowest sixtile of contacts. The same neighborhood is instead in the top sixtile of price per m2, together with other OMI microzones in the center. A similar pattern can be seen in Milan: the popular neighborhoods of Duomo and Brera (indicated by the arrow) are in the lowest sixtile of clicks, but in the top sixtile of price per m2.

So both in Rome and Milan prices are highest in the center and decrease towards the peripheries, but online interest is maximal in an intermediate area between the center and the peripheries. We conjecture that this pattern may be related to income and wealth inequality. Only few people in the top of the income/wealth distribution can afford—and so look for—apartments in the center of Rome and Milan, where the prices are easily above 6000 euros per m2. This results in a lower number of clicks and contacts with respect to neighborhoods that are still attractive but less expensive. It is also interesting that the neighborhoods of Sapienza University in Rome and of Polytechnic and Statale University (Città Studi) in Milan are in the top sixtile of daily contacts/clicks per ad, suggesting a high demand from students.

## Evidence that online interest proxies demand

Is online interest a good proxy for actual demand? In this section we provide evidence that supports this hypothesis, showing that online interest has the same effect of demand on time on market, liquidity and prices. We run our analysis both at the level of individual ads and aggregating data over OMI microzones/cities. We mostly follow the microeconometrics literature [24, 34], in that we assess whether the effect of clicks/contacts is statistically significant by running hypothesis tests on the coefficients of pre-specified statistical models. The alternative would be, as in machine learning, to perform model selection and estimation jointly, but in this way the estimated parameters may not indicate any structure due to the correlation among predictors [29, 35]. We assume linear relations among variables, which is certainly a restriction but makes it possible to give a simple interpretation to the coefficients of these linear models. In addition, we can control in a transparent way for other characteristics that clicks and contacts may also be a proxy for (e.g. intrinsic quality of a dwelling/neighborhood).

### Evidence at the individual level

We test whether high online interest for a dwelling is correlated with shorter time on market and with price revisions. We only focus on ads that have been posted since the beginning of 2016 and that have no duplicates (which would bias the analysis).^4^

We construct two variables, *RELCLICKS* and *RELCONTACTS*, to quantify the relative interest in a particular dwelling with respect to all other dwellings in the same OMI microzone. In the case of time on market, *RELCLICKS* and *RELCONTACTS* are defined as the total number of clicks/contacts on an ad, divided by the average number of clicks/contacts in the corresponding OMI microzone during the same period in which the ad has been online. In the case of price revisions, this definition would not work. Indeed, price revisions trigger a change of online interest, as can be seen in Fig. 3D. This would lead to a dubious interpretation of the results. To solve this problem, when analyzing price revisions we define *RELCLICKS* and *RELCONTACTS* as the ratio of clicks/contacts in the first 14 days since the ad was posted to the average of the OMI microzone in the same period. (We also discard ads that had a price revision within 15 days.) This choice is justified by the peak of clicks/contacts in the first few days after ads are posted (Fig. 3A–B).

We start studying the effect of clicks and contacts on time on market. We define the variable *TIMEONMARKET* as the difference between the dates in which an ad was removed and the same ad was uploaded. We are interested in whether there exists a robust statistical association among these variables, so we control for other quantities that may also have an effect on time on market. In particular, we run an Ordinary Least Squares (OLS) regression on the following model:

1 $$ \log y_{i} = \alpha + \beta \log x_{i} + \boldsymbol{ \gamma} \boldsymbol{z}_{i} + u_{i}, $$

where $y_{i}$ is the dependent variable and corresponds to *TIMEONMARKET* for ad *i*, $x_{i}$ is the variable of interest and corresponds to *RELCLICKS* or *RELCONTACTS*, $\boldsymbol{z}_{i}$ is a vector of control variables and $u_{i}$ is a residual term. Differentiating Eq. (1) we get

2 $$ \frac{\delta y_{i}}{y_{i}} = \beta \frac{\delta x_{i}}{x_{i}} + \boldsymbol{\gamma} \frac{\delta \boldsymbol{z}_{i}}{\boldsymbol{z}_{i}}. $$

This justifies the standard microeconometric interpretation of “log–log” regressions [34]: the coefficient *β* represents an *elasticity*, that is the relative change of the variable *y* in response to a relative change of the variable *x*, holding the control variables constant (*ceteris paribus*). This can be interpreted either as a correlation (a *β*% change in *y* is associated with a 1% change in *x*), or causally (had $x_{i}$ been 1% different, $y_{i}$ would have been *β*% different). The causal interpretation holds if all possible control variables are included in $\boldsymbol{z}_{i}$, if there is no reverse causality from *y* to *x* and if there is no measurement error [34]. In this section we do not interpret the coefficients causally, while we focus on causality in Sect. 5.2.

In Table 1, columns (1)–(2), we see the results from running the OLS regression in Eq. (1). The control variables are *RELPRICEM*2, defined as the ratio of the price per m2 of the dwelling to the average price per m2 in the OMI microzone in the same period in which the ad was posted; physical characteristics of the dwelling, including *FLOORAREA*, maintenance *STATUS* and *ROOMS*; and OMI microzone and quarter dummies, to capture spatial and temporal effects. (Note that by considering these control variables, we are estimating more than 1400 coefficients, mostly due to the large number of OMI microzone dummies.)

**Table 1:** Effect of online interest on time on market and chance of price revisions

	*Dependent variable:*			
	*LOGTIMEONMARKET*		*PRDECREASE*	*PRINCREASE*
	*OLS*		*logistic*	*logistic*
	(1)	(2)	(3)	(4)
*RELCLICKS*	−0.520^∗∗∗^		−0.095^∗∗∗^	0.156^∗∗∗^
	(0.004)		(0.013)	(0.042)
*RELCONTACTS*		−0.481^∗∗∗^		
		(0.004)		
*RELPRICEM*2	−0.060^∗∗∗^	−0.022	0.222^∗∗∗^	−0.375^∗∗∗^
	(0.010)	(0.014)	(0.017)	(0.023)
*FLOORAREA*	0.0002^∗∗∗^	−0.0003^∗∗∗^	0.0001	−0.0002
	(0.0001)	(0.0001)	(0.0001)	(0.0003)
*STATUS*	−0.033^∗∗∗^	0.007	−0.203^∗∗∗^	0.539^∗∗∗^
	(0.004)	(0.005)	(0.008)	(0.028)
*ROOMS*	0.024^∗∗∗^	0.012^∗∗^	−0.028^∗∗∗^	0.016
	(0.004)	(0.006)	(0.007)	(0.025)
Constant	3.853^∗∗∗^	4.332^∗∗∗^	1719.883^∗∗∗^	1559.833
	(0.364)	(0.111)	(639.811)	(7900.740)
Observations	71,221	26,536	128,829	128,829
Adjusted R-squared	0.327	0.457	/	/
Residual deviance	/	/	141,916	18,944
AIC	174,083	56,598	145,972	21,848

*Note:* ^∗^*p*<0.1; ^∗∗^*p*<0.05; ^∗∗∗^*p*<0.01. In the model diagnostics, adjusted R-squared only applies to OLS, while residual deviance only applies to logistic regression. Additional controls: OMI microzone and quarter dummies. *RELCLICKS* and *RELCONTACTS* are in logs. In columns (1)–(2) *RELCLICKS* and *RELCONTACTS* are calculated over the entire lifespan of the ad. Instead in (3)–(4) *RELCLICKS* is calculated over the first 14 days from upload. The results for *RELCONTACTS* are similar and given in the text.

The coefficient on *RELCLICKS* is highly statistically significant and can be interpreted in the following way: a 1% higher number of clicks is on average associated with a 0.52% shorter time on market, holding all control variables constant. Here we cannot interpret this coefficient causally because e.g. the time on market influences the relative number of clicks, as the temporal profile of clicks is not uniform (Fig. 3), and so there is reverse causality [34]. The elasticity for the variable *RELCONTACTS* is similar.^5^ Looking at the control variables, it appears that dwellings with higher relative price stay shorter on the market, although in this case statistical significance is less clear.

To quantify variable importance in the determination of the time on market we use a regression tree. In particular, we use the R package rpart and select the hyperparameters for visualization purposes. We use the same variables *RELCLICKS*, *RELCONTACTS*, *RELPRICEM*2, *FLOORAREA*, *STATUS*, *ROOMS* as in Table 1. Instead of controlling for location using the fine-grained OMI microzone dummies, we use distance from the center and city dummies, and only consider ads in the four largest cities (Rome, Milan, Naples and Turin). This again is dictated by the necessity to produce a discernible regression tree.

**Figure 7:**
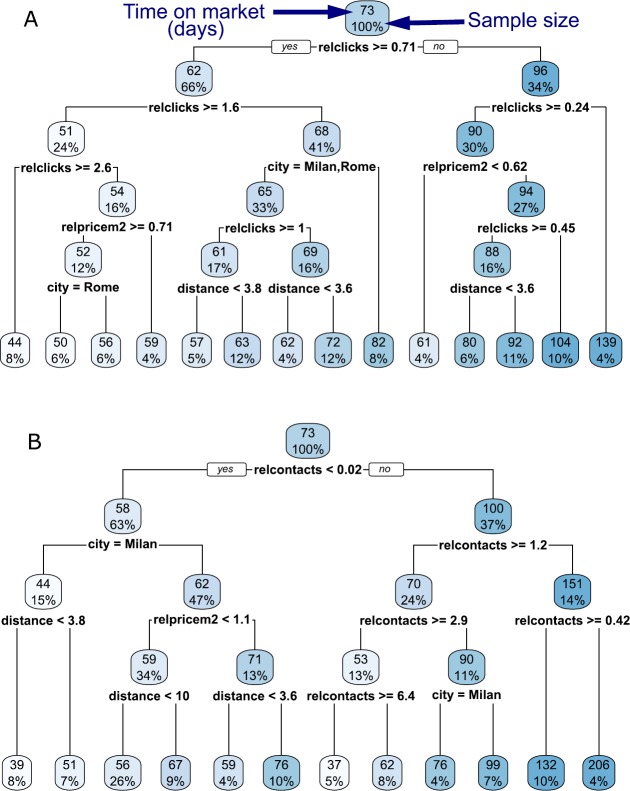
Regression trees quantifying variable importance in the determination of the time on market. For each node, the number on top is the average time on market (in days), and the percentage below is the sample size in that node. Nodes are colored in darker shades of blue for longer time on market. From each node, the edge to the left is a TRUE to the condition, while the edge to the right is a FALSE. (**A**) The relative number of clicks is by far the most important variable because it determines the first splits. It is followed by geographical and price variables. Higher number of clicks (edges to the left) predicts shorter time on market. (**B**) The first split distinguishes between ads that received no contacts (left branch), and ads that received at least one (right branch). In the right branch, contacts are the most important variable. The figure has been generated using R package rpart.plot

In Fig. 7A we consider *RELCLICKS* as a measure of online interest. We can see that the first two splits are based on whether *RELCLICKS* is larger than a certain threshold, and that higher values of *RELCLICKS* lead to shorter time on market in the leaf nodes. At the third split it is also possible to see a splitting decision based on geographical characteristics—whether the dwelling is in Rome or Milan, or whether it is in Naples or Turin—and on the relative price per m2. In Fig. 7B we instead consider *RELCONTACTS* as the measure of online interest. In this case a potential problem is that 2/3 of the ads received no contacts (see Sect. 3.1). For all of these ads $\mathit {RELCONTACTS}=0$, and so the variable *RELCONTACTS* cannot be used to determine the time on market. This can be seen from the left branch of the tree in Fig. 7B, as the first split effectively distinguishes between the ads that receive no contacts and the ads that received at least one. In the right branch, *RELCONTACTS* is by far the most important variable, and a higher number of contacts leads to shorter time on market in the leaf nodes.

After testing that online interest is predictive of time on market, we now test whether it is predictive of price revisions. In our dataset, about 25% of the dwellings had a price change; out of these, about 6% had an increase in price, and the complementary 94% had a price decrease. These figures are consistent with data from the Italian Housing Market Survey (jointly run by Banca d’Italia), showing that the share of transactions in which the actual transaction price was equal or higher than the asking price was about 3.0% in 2015, 5.1% in 2016 and 5.6% in 2017. However, there are two caveats that we should make here. First, these price revisions do not necessarily reflect the transaction price (other revisions may occur during offline bargaining). Second, we cannot know why price revisions occurred. In particular, in the case of price increases, this may reflect an auction, but also the fact that the agency corrected a wrong posted price. Yet, our imperfect measures for price revisions carry information, and so it is useful to see what the effect of online interest is on them.

In Table 1, column (3), we show the results from running a logistic regression on the binary variables *PRDECREASE*, taking value 1 if the price of the dwelling was revised downward, and 0 if it was not revised or if it was revised upward. In column (4) we consider the variable *PRINCREASE*, defined as *PRDECREASE* but equal to 1 if the price was revised upward, and 0 otherwise. With logistic regressions, the interpretation of the coefficients is less straightforward than with OLS.^6^ It is first necessary to take exponentials. Doing this, looking at the coefficient on *RELCLICKS* in Table 1 we get $\exp (-0.095)= 0.91$ in the case of *PRDECREASE*, and $\exp (0.156)=1.17$ in the case of *PRINCREASE*. These numbers can then be interpreted as changes in the *odds ratio*, that is the ratio of the probability that the event happens to the complementary probability that it does not happen. Given the logarithmic transformation of *RELCLICKS*, the interpretation is that a 1% increase in the relative number of clicks is associated with a 0.09% reduction in the odds of a downward price revision, and with a 0.17% increase in the odds of an upward price revision.

The results for *RELCONTACTS* are similar, except that we cannot make a logarithmic transformation of this variable because the condition $\mathit {RELCONTACTS}>0$ is satisfied by too few ads with at least one price revision. To deal with this, we run a regression akin to Eq. (1), but in which the dependent variable *RELCONTACTS* enters linearly. The coefficients are −0.028 (0.002)^∗∗∗^ for *PRDECREASE*, and $0.012\ (0.003)^{***}$ for *PRINCREASE*. Applying the same method as above, the interpretation for these coefficients is that a *unit* increase in *RELCONTACTS* is associated with a 0.03% reduction in the odds of a downward price revision, and with a 0.01% increase in the odds of an upward price revision.

In Table 2 we go one step further and we investigate the effect of online interest on the *magnitude* of price revisions. We define $\mathit {PRICEVAR}=(p_{2}-p_{1})/p_{1}$, where $p_{1}$ is the initial price and $p_{2}$ is the revised price. Considering the relative price change is necessary to make this variable scale independent—some price changes are much bigger than others in absolute terms. We also define *PRICEVAR*− (*PRICEVAR*+) as the absolute value of *PRICEVAR*, but only for the ads with a downward (upward) price revision. We see that a 1% increase in the relative number of clicks leads to a 0.009% increase in price,^7^ and that magnitudes are similar when breaking down the regression in positive and negative price changes. Note that these coefficients are much smaller than the equivalent ones in the case of the time on market (Table 1, columns (1)–(2)). A regression tree (not reported) confirms that *RELCLICKS* is not as important in determining price changes as it was in determining the time on market. The results for *RELCONTACTS* are again similar to those for *RELCLICKS*, with the difference that *RELCONTACTS* enters linearly in the regression for the same reasons explained in the paragraph above. We find that *RELCONTACTS* has a coefficient of 0.00060 (0.00007)^∗∗∗^ on *PRICEVAR*, of $-0.00023\ (0.00006)^{***}$ on *PRICEVAR*−, and of $0.00052\ (0.00030)^{*}$ on *PRICEVAR*+.

**Table 2:** Effect of online interest on the magnitude of price changes

	*Dependent variable:*		
	*PRICEVAR*	*PRICEVAR*−	*PRICEVAR*+
	(1)	(2)	(3)
*RELCLICKS*	0.009^∗∗∗^	−0.007^∗∗∗^	0.008^∗∗^
	(0.001)	(0.001)	(0.004)
*RELPRICEM*2	0.001	−0.006^∗∗∗^	−0.040^∗∗∗^
	(0.001)	(0.001)	(0.006)
*FLOORAREA*	−0.00004^∗∗∗^	0.00003^∗∗∗^	−0.00003
	(0.00001)	(0.00000)	(0.00004)
*STATUS*	0.011^∗∗∗^	−0.007^∗∗∗^	−0.001
	(0.0004)	(0.0003)	(0.002)
*ROOMS*	0.005^∗∗∗^	−0.005^∗∗∗^	−0.005^∗∗^
	(0.0004)	(0.0003)	(0.002)
Constant	−4.725^∗∗∗^	6.717^∗∗∗^	2.777
	(1.680)	(1.382)	(10.226)
Observations	36,344	34,552	1792
Adjusted R-squared	0.075	0.089	0.127
AIC	−100,790	−111,020	−4460

*Note:* ^∗^*p*<0.1; ^∗∗^*p*<0.05; ^∗∗∗^*p*<0.01. Additional controls: OMI microzone and quarter dummies. *RELCLICKS* and *RELPRICEM*2 are in logs. *RELCLICKS* is calculated over the first 14 days from the upload of the ad. The results for *RELCONTACTS* are similar and given in the text.

### Evidence at the aggregate level

We aggregate data over OMI microzones and cities and test whether aggregate online attention is a leading indicator of liquidity and prices. We mostly follow the approach of van Dijk and Francke [22], who analyze a dataset of online housing ads in the Netherlands and show that the average number of clicks Granger-causes liquidity and prices. We confirm their findings, and extend their analysis by considering smaller geographical aggregates—the spatial unit in their analysis [22] is municipalities, here we also consider OMI microzones—and contacts in addition to clicks.^8^

Our underlying hypothesis is that a tight market—that is, a market with relatively high demand as compared to the supply—at time *t* predicts an increase in price and liquidity at time $t+1$, where *t* is an arbitrary temporal unit. (In this paper *t* corresponds to quarters, see below.) This can be justified theoretically in various ways. Carrillo *et al.* [18] use a search model in which a demand shock occurs. Sellers’ expectations on the number of buyers adjust slowly, and therefore it takes time to reach a different equilibrium. We empirically test the hypothesis that an increase in clicks leads to a lagged increase in liquidity and prices, using OMI microzones or cities as spatial units, and quarters as temporal units.

We define the following variables (for all combinations of spatial units and temporal units):

- *LOGLIQUIDITY* is the logarithm of the ratio between the number of dwellings removed from the dataset and the number of dwellings for sale. In Ref. [33] we show that the number of dwellings removed from the dataset is highly correlated to the number of actual sales, as measured from OMI. (A dwelling is a cluster of duplicate ads. If we did not deal with duplicates, measures of liquidity would be biased [33].)
- *LOGPRICEM*2 is the logarithm of the average price per m2. In Ref. [33] we show that the price per m2 calculated from this dataset is highly correlated to the price per m2 calculated by OMI using actual transactions.
- *LOGCLICKS* and *LOGCONTACTS* are the logarithms of the average number of clicks/contacts per ad.

As control variables, we first of all consider *LOGLIQUIDITY* and *LOGPRICEM*2 lagged by one temporal unit, to avoid the possibility that lagged *LOGCLICKS* and *LOGCONTACTS* are a proxy for these variables. Other control variables are city and quarter dummies. In addition, we use socio-economic characteristics from the 2011 census (see Sect. 2). These are *DEGREE*, i.e. the fraction of people with a university degree; *UNEMPLOYED*, i.e. the unemployment rate; *OWNEDHOUSES*, i.e. the fraction of owned houses, as opposed to rented properties; and *FOREIGN*, i.e. the percentage of foreign population.

In Table 3 we test whether lagged online interest is predictive of liquidity. We consider two time lags (quarters) for our measures of online interest, and also control for liquidity in the previous quarter. In columns (1) and (2) we consider OMI microzones as spatial units; in columns (3) and (4) the spatial units are cities. In all cases except column (2), the coefficients on the measures of online interest are positive and statistically significant at a two quarters lag; in the case of column (2), the coefficient on $\mathit {LOGCONTACTS}_{t-2}$ is only statistically significant at the 10% level, while the coefficient on $\mathit {LOGCONTACTS}_{t-1}$ is more significant. Table 4 shows the results when the dependent variable is the price per m2. The results are similar, although the coefficients on the variables defining online interest are smaller and not significant in column (3).

**Table 3:** Lagged effect of aggregate online interest on liquidity

	*Dependent variable:*			
	$\mathit {LOGLIQUIDITY}_{t}$			
	OMI micro-zone		City	
	(1)	(2)	(3)	(4)
$\mathit {LOGLIQUIDITY}_{t-1}$	0.230^∗∗∗^	0.230^∗∗∗^	0.488^∗∗∗^	0.384^∗∗∗^
	(0.019)	(0.019)	(0.043)	(0.047)
$\mathit {LOGCLICKS}_{t-1}$	0.060		−0.088	
	(0.037)		(0.076)	
$\mathit {LOGCLICKS}_{t-2}$	0.146^∗∗∗^		0.194^∗∗^	
	(0.037)		(0.078)	
$\mathit {LOGCONTACTS}_{t-1}$		0.075^∗∗∗^		0.039
		(0.019)		(0.059)
$\mathit {LOGCONTACTS}_{t-2}$		0.037^∗^		0.184^∗∗∗^
		(0.019)		(0.062)
*DEGREE*	−0.574^∗∗∗^	−0.483^∗∗∗^	0.332	−0.374
	(0.114)	(0.111)	(0.533)	(0.531)
*UNEMPLOYED*	0.752	0.861	1.969^∗^	1.547
	(0.647)	(0.647)	(1.151)	(1.109)
*OWNEDHOUSES*	−0.116	−0.093	−0.778^∗^	−0.392
	(0.095)	(0.095)	(0.437)	(0.423)
*FOREIGN*	−0.214	−0.314^∗∗^	0.964	−0.188
	(0.133)	(0.133)	(0.688)	(0.652)
Constant	−2.400^∗∗∗^	−1.013^∗∗∗^	−1.526^∗∗∗^	−0.859^∗∗^
	(0.208)	(0.135)	(0.576)	(0.350)
Fixed effects	City + quarter	City + quarter	Quarter	Quarter
Observations	2977	2977	423	420
Adjusted R-squared	0.372	0.370	0.350	0.359
AIC	1243	1249	336	301

*Note:* ^∗^*p*<0.1; ^∗∗^*p*<0.05; ^∗∗∗^*p*<0.01.

**Table 4:** Lagged effect of aggregate online interest on price

	*Dependent variable:*			
	$\mathit {LOGPRICEM}2_{t}$			
	OMI micro-zone		City	
	(1)	(2)	(3)	(4)
$\mathit {LOGPRICEM}2_{t-1}$	0.511^∗∗∗^	0.511^∗∗∗^	0.639^∗∗∗^	0.619^∗∗∗^
	(0.014)	(0.014)	(0.039)	(0.041)
$\mathit {LOGCLICKS}_{t-1}$	0.035		−0.050	
	(0.025)		(0.058)	
$\mathit {LOGCLICKS}_{t-2}$	0.051^∗∗^		−0.040	
	(0.026)		(0.060)	
$\mathit {LOGCONTACTS}_{t-1}$		0.019		−0.026
		(0.013)		(0.046)
$\mathit {LOGCONTACTS}_{t-2}$		0.030^∗∗^		0.100^∗∗^
		(0.013)		(0.050)
*DEGREE*	0.836^∗∗∗^	0.866^∗∗∗^	0.938^∗∗^	0.513
	(0.083)	(0.081)	(0.414)	(0.424)
*UNEMPLOYED*	−2.736^∗∗∗^	−2.694^∗∗∗^	−0.298	−0.897
	(0.456)	(0.455)	(0.878)	(0.897)
*OWNEDHOUSES*	−0.503^∗∗∗^	−0.493^∗∗∗^	−0.392	−0.057
	(0.068)	(0.067)	(0.333)	(0.336)
*FOREIGN*	−0.444^∗∗∗^	−0.483^∗∗∗^	0.109	0.107
	(0.094)	(0.093)	(0.524)	(0.519)
Constant	3.366^∗∗∗^	3.945^∗∗∗^	3.390^∗∗∗^	2.921^∗∗∗^
	(0.171)	(0.148)	(0.556)	(0.417)
Fixed effects	City + quarter	City + quarter	Quarter	Quarter
Observations	2977	2977	423	420
Adjusted R-squared	0.835	0.835	0.540	0.541
AIC	−919	−920	110	109

*Note:* ^∗^*p*<0.1; ^∗∗^*p*<0.05; ^∗∗∗^*p*<0.01.

## Duplicates, demand and prices

The main problem of this dataset is the substantial fraction of duplicate ads. We devise a machine learning algorithm to identify duplicates and to cluster ads so that each cluster corresponds to a unique dwelling. This *deduplication* procedure was necessary to clean the data for the analysis in the previous sections. In this section we show that duplicates can also be exploited to shed some light on a classical problem in econometrics: what difference in demand is caused by a difference in price?

### Description of the deduplication algorithm

We adapt standard methodologies for the deduplication of datasets [36, 37] to our specific case. Here we only give an overview of the working of the algorithm. For a more detailed description and the pseudocodes, see Ref. [33].

*Model*. We perform a pairwise comparison, meaning that we compare each ad with all other ads that are close enough—both in terms of geographical coordinates and price—to potentially be duplicates. We use a C5.0 classification tree. For each pair of ads the classification tree outputs a probability that they are duplicates. If this probability is larger than 0.5, we consider the two ads as duplicates. We implement two different C5.0 models, depending on whether the ads are posted by the same agency or not.

*Predictors*. Among the predictors we consider the geographical distance, the difference in price, the temporal distance between the upload dates, and the difference between the physical characteristics of the dwellings. As some physical characteristics are categorical variables, we consider different degrees of similarity, taking advantage of the natural order of the classes. For example, two ads with reported maintenance status “new” and “good” respectively are more likely to be duplicates than two ads with “new” and “to renovate”. A final important predictor is the distance between the textual description of the two ads. For this variable we consider two different measures, depending on whether the ads are posted by the same agency or not. In the first case we use the Levenshtein distance, as only a few words may have changed.

In the case of different agencies, we instead compute the cosine similarities between the vectors produced using the *doc2vec* algorithm [38], as implemented in gensim [39]. Doc2vec is an unsupervised algorithm that learns vector representations of documents, so that two documents that are close in “context” are also close in vector space. We use the Distributed Memory version of doc2vec. This is a two-layer neural network in which the output neuron is a word *w* and the input neurons are a set of words surrounding *w* and an identifier for each document. Learning occurs by minimizing the distance between the predicted and actual *w*, over all *w* and all documents. We choose the training settings (number of training epochs, use of stopwords, minimum frequency of words, etc.) via cross-validation. In particular, we check how often the out-of-sample predicted vector for a document is closest to the in-sample learned vector for that document. In the best performing case, this is achieved 85% of the times.

*Training*. We manually construct a training sample by verifying the photos of the ads on the website. The training sample for the ads of different agencies is made up of 9997 pairs of ads; among them 3483 are duplicates (true positive, TP). The training sample for the ads of the same agency is made up of 8688 observations and 1473 are duplicates.

In order to assess the performance of the two models we randomly split the training sample in two different sub-samples: the first one (90% of the observations) is used to estimate the models using *boosting*, the second one (10% of the observations) is used for the out-of-sample assessment of the classification performance. We repeat the operation 1000 times (drawing different sub-samples) and we evaluate the performance based on average results. Since the number of true negatives (ads that are not duplicates and are identified as such) is much larger than the number of true positives, using the classic accuracy rate can be misleading. For this reason we consider measures of classification performance that do not rely on the number of true negatives, namely: precision, recall and F-measure.

We show the results in Table 5. As expected, the model for ads of the same agency is significantly more precise than the one for ads of different agencies. Ads posted from the same agency and related to the same dwelling have almost all the characteristics in common, therefore it is easier to identify them. However, as the F-measure is equal to 0.907, also the classification performance of the C5.0 model for ads of different agencies is quite good.

**Table 5:** Assessment of C5.0 models

	Observations	Duplicates	Precision	Recall	F-measure
Different agency	9997	3483	0.923	0.892	0.907
Same agency	8688	1473	0.952	0.963	0.957

Precision = TP/(TP + FP). Recall = TP/(TP + FN). F-measure = 2 ∗ (Precision ∗ Recall)/(Precision + Recall). TP = true positive; FP = false positive; FN = false negative.

**Figure 8:**
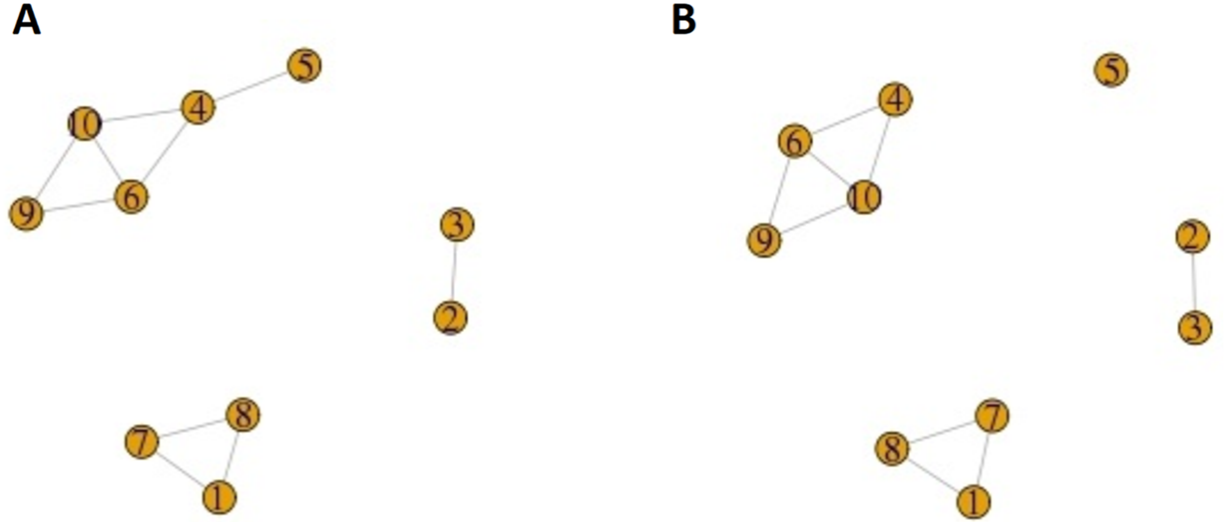
Example of the clustering algorithm. (**A**) Initial situation. Each node is an ad; nodes are linked if they are duplicates. The 1–7–8 and 2–3 subgraphs are fully connected; the 4–5–6–9–10 subgraph is not. (**B**) Final situation. We split the cluster 4–5–6–9–10 by removing the edge with minimal duplicate probability, in this case 4–5. The cluster 4–6–9–10 is still not fully connected, but sufficiently connected. As a condition for connectivity, we choose $M\geq \frac{5}{6}\frac{N(N-1)}{2}$, where *M* is the number of edges and *N* the number of nodes in the cluster

*Clustering*. From pairs of ads that are duplicates, we have to create clusters of ads that refer to the same dwellings. We decide whether a cluster of ads refers to the same housing unit based on a measure of connectivity of the cluster, illustrated in Fig. 8.

Once we have created the clusters of ads identifying different dwellings, we collapse the information contained in multiple ads related to the same dwelling. As a general rule, for each characteristic we take the one with highest absolute frequency.

*Real time implementation*. To make the methodology computationally feasible, we apply an iterative approach. In particular, we process the ads progressively as soon as they are published on the website. In this way we are able to reduce the number of pairwise comparisons between ads.

### Estimating the price elasticity of demand

It is a typical problem for businesses to forecast demand. But often companies need to understand how demand would be different if the price of their product was different. This is a causal question, and it is a much trickier task. Demand and supply are simultaneously determined, and to identify demand or supply curves it is often necessary to look for exogenous “shifters” [24]. Here we propose a method to estimate the *price elasticity of demand*—the relative difference in demand caused by a relative difference in price—by exploiting duplicate ads. We stress that here we use a proxy of demand (the number of clicks), differently from most of the literature that considers realized demand (for example, Cohen *et al.* [26] use actual Uber rides purchased by users of the Uber app).

Our method is inspired by the potential outcomes framework [31], and in particular by propensity score matching [32]. A typical way to assess causality is to assign some treatment to a set of randomly chosen units, and then compare the effects on units that received treatment vs. units that did not receive it. This does not work in observational studies in which units decide if they want to undergo treatment. The basic idea of propensity score matching is to compare units that are very similar except for their choice to receive treatment. Our method compares units that are *identical*, except for the “treatment” variable.

Indeed, our strategy to compute the elasticity of demand is to consider pairs of duplicate ads posted with a different price. This difference in price may simply reflect the decision of the seller to revise the price jointly with the decision of the agency to post a new ad for the same dwelling, or it could be that different agencies suggest different prices. Our key assumption is that all differences in the number of clicks between the two ads can only be imputed to the differences in price. Indeed, the two ads should have identical or very close characteristics, so that the only difference in the number of clicks should come from the user preference for cheaper dwellings—or from one of the two prices exceeding her maximum willingness to pay.

The full dataset contains 113,365 pairs of duplicate ads. We restrict our sample to ads published after the beginning of 2016, and to pairs for which the two ads have been posted within 60 days from each other. We finally remove ads whose price changed in the period of observation, because a price change makes one of the two ads different from the other. This selection leaves us with 16,824 ads, or equivalently 8412 unique dwellings.

We define $c_{1i}$ as the normalized number of clicks on ad 1 of duplicate pair *i*. This is calculated as $c_{1i}=C_{1i}/C_{1i}^{\mathrm {OMI}}$, where $C_{1i}$ is the total number of clicks on ad 1 in the first 14 days from upload and $C_{1i}^{\mathrm {OMI}}$ is the average number of clicks on the ads of the OMI microzone in the same period. (We focus on the first 14 days to deal with some of the caveats that are described below.) The definition of $c_{2i}$ for ad 2 is identical. The division by $C_{1i}^{\mathrm {OMI}}$ and $C_{2i}^{\mathrm {OMI}}$ is needed to control for different demand conditions at the times the two ads were posted. We also define $p_{1i}$ and $p_{2i}$ as the prices of ads 1 and 2 in the 14 days of observation. We consider the regression

3 $$ \Delta c_{i} = \alpha + \beta \Delta p_{i} + u_{i}, $$

where *α* is the intercept, *β* is the price elasticity of demand, $u_{i}$ is a residual term and the dependent and independent variables are respectively

4 $$ \Delta c_{i} = \frac{c_{1i}-c_{2i}}{(c_{1i}+c_{2i})/2} \quad \text{and} \quad \Delta p_{i} = \frac{p_{1i}-p_{2i}}{(p_{1i}+p_{2i})/2}. $$

Running the regression (3) on the 8412 pairs of ads we estimate $\beta =-0.657\ (0.048)^{***}$ and $\alpha =-0.012\ (0.007)^{*}$ (${}^{*}p<0.1$; ${}^{**}p<0.05$; ${}^{***}p<0.01$). As expected, the price elasticity of demand is negative and highly significant, while the intercept is only marginally significant (at the 10% level). Here *β* has the causal interpretation of an elasticity: a 1% higher price causes on average a 0.66% lower number of clicks relative to the average in the OMI microzone.

As a robustness test, we also check that our results are robust to a different measure of $c_{1i}$ and $c_{2i}$. In particular we consider the number of clicks in the first 7 or 10 days, to deal with the potential issue that both duplicate ads may be online at the same time. With 7 days we estimate $\beta =-0.608\ (0.044)^{***}$ and $\alpha =0.004\ (0.006)$; with 10 days we find $\beta =-0.633\ (0.044)^{***}$ and $\alpha =0.004\ (0.006)$. This confirms the results, although the elasticities are slightly smaller than in the 14 days case. In addition, here the intercept is not statistically significant even at the 10% level.

Our identification strategy (in the econometrics jargon, it means technique to assess causality) comes with a series of caveats. First, users of the website should not be able to identify duplicates before clicking on them. We think this is reasonable in most cases. Indeed, if users search by list, duplicate ads may be listed far from each other and potentially have different “front pictures”. And if users search by map, it is quite common that multiple dwellings in the same block of flats are sold at the same time, so users should not be able to disambiguate between duplicate ads and multiple dwellings in the same block. Our choice of focusing on the first 14 days makes it unlikely that users decide not to re-click on ads if they realized they were duplicates.

The second caveat is that agencies should be assigned randomly to the ads within the pair. Indeed, agencies can pay to upload “premium ads”, which are shown high up in the list and so receive a higher number of clicks. If agencies are systematically more likely to upload premium ads for more (or less) expensive ads, our estimates can be inconsistent. Third, small differences in characteristics should be assigned randomly to the ads within the pair (i.e., differences are just due to reporting errors). Fourth, the deduplication algorithm has a low rate of false positives, that is pairs of ads that are identified as duplicates but are not so. (However, in this case one could argue that if the machine learning algorithm identifies the ads as duplicates, probably they are so similar that our identification strategy should work as well.) Although at least some of these effects are probably present to some extent, we think that they could alter the value of the estimated elasticity by at most some decimal points.

## Conclusion

In the last few years a growing amount of research has used data coming from online sources to analyse the housing market (further to the references listed so far, see also Refs. [40–44]). The large number of housing ads websites—including Zillow.com and Trulia.com in the U.S., Zoopla.co.uk in the U.K., Immobilienscout24.de in Germany, Funda.nl in the Netherlands, etc.—will probably further increase the interest of researchers in this type of data. To the best of our knowledge, our work is the first to characterize online interest for individual ads.

We describe the distribution and temporal profile of two measures of online interest, clicks on ads and uses of the contact form on the page of each ad. We show that both the distributions of the total number of clicks and of the total number of contacts are heavy tailed, and that a peak of clicks/contacts occurs in the first few days since an ad was posted. We then use inferential statistics to provide evidence that online interest indeed proxies demand. Ads that receive a high number of clicks/contacts relative to other ads in the same neighborhood stay shorter online, it is less likely that the price is revised downward, and more likely that it is revised upward. We also aggregate data at the level of neighborhoods and cities, replicating existing results in the literature that document a lagged increase in prices and volume of transactions that follows a spike in demand. As time on market, price revisions and liquidity respond to online interest in the same way as to actual demand, we deduce that clicks and contacts are a good proxy.

Our second key contribution is to show how these data can be used to estimate the price elasticity of demand, the relative change in demand in response to a relative change in price. This should be intended in the sense of a thought experiment—had the price been different by *x*%, demand would be different by *y*%—and not in the sense of revising the price when the ad was already online. We exploit the substantial fraction of duplicate ads identified with a machine learning algorithm. Under some caveats, differences in demand between two ads that advertise the same dwelling can only be caused by differences in price (controlling for the different times at which the two ads may be posted). Quantitatively, we show that a 1% higher price causes a 0.66% lower number of clicks.

Econometrics is mostly used to understand causality, while machine learning is mostly used for prediction. It has recently been argued that the strengths of the two approaches should be combined [28–30]. For example, Belloni *et al.* [45, 46] suggest to use LASSO to select among a large number of instrumental variables. The method we introduce here is less general as it relies on the specific existence of duplicate ads with different price, but it combines ideas from supervised machine learning and from the potential outcomes framework [31, 32]. As housing and non-housing marketplace websites are attracting increasing interest from researchers, we think that it can be applied in other circumstances. For example, it would be interesting to apply the method on “classifieds” (classified advertisement) websites such as Craiglist.org, Gumtree.com, Ganji.com, Leboncoin.fr, Subito.it, etc.

## Footnotes

1 We have intentionally avoided to use the term demand *estimation* as it is sometimes used to indicate causal identification and sometimes to refer to demand forecasting.

2 Using OMI microzones as spatial units is not necessarily an optimal choice, and it is known that some results may in general depend on how spatial units are constructed [47]. Yet, these microzones are constructed to estimate the market value of properties for tax purposes, so it is reasonable to assume that boundaries are drawn meaningfully. An interesting extension of this work would be to use clustering methods to construct data-driven spatial units.

3 Due to representativeness concerns, we remove data corresponding to the bottom 5% of the distribution of the number of visible ads. In a few small cities and for specific quarters only a few ads are visible, and these are often outliers.

4 For the analysis of the time on market, we only consider ads that have been removed from the dataset as this ensures that we follow the ad throughout its lifespan.

5 In the regression in column (2) we have only considered the ads that had at least one contact, otherwise $\mathit {RELCONTACTS}=0$ and the logarithmic specification would not be possible.

6 See Ref. [24] for a textbook treatment, or the clear explanation at https://stats.idre.ucla.edu/other/mult-pkg/faq/general/faq-how-do-i-interpret-odds-ratios-in-logistic-regression/.

7 There is no need to take a logarithmic transformation of *PRICEVAR*, as it is already defined as a relative change.

8 From a technical point of view, our analysis differs from van Dijk and Francke [22] in that we consider the *levels* of clicks, contacts, liquidity and prices, whereas they consider *first differences*. Our temporal span of 1.5 years (2015 data cannot be used here) does not make it possible to remove seasonality, impairing an analysis based on first differences.

## Abbreviations

OMI: Osservatorio del Mercato Immobiliare (Real-Estate Market Observatory)
OLS: Ordinary Least Squares
TP: True Positives
TN: True Negative
FN: False Negative
LASSO: Least Absolute Shrinkage and Selection Operator
